# A Comprehensive Assessment of the Effects of Transgenic Cry1Ac/Cry1Ab Rice Huahui 1 on Adult *Micraspis discolor* (Fabricius) (Coleoptera: Coccinellidae)

**DOI:** 10.1371/journal.pone.0142714

**Published:** 2016-02-25

**Authors:** Xia Zhou, Yunling Guo, Hua Kong, Jiao Zuo, Qixing Huang, Ruizong Jia, Anping Guo, Lin Xu

**Affiliations:** Key laboratory of Biology and Genetic Resources of Tropical Crops, Ministry of Agriculture, Institute of Tropical Bioscience and Biotechnology, Chinese Academy of Tropical Agriculture Science, Haikou, Hainan, 571101, P.R. China; Instituto de Biotecnología, Universidad Nacional Autónoma de México, MEXICO

## Abstract

*Micraspis discolor* (Fabricius) (Coleoptera: Coccinellidae) is a widely distributed coleoptera predator in southern Asia in rice ecosystem, and adult *M*. *discolor* feed on both rice pollen and soft-bodied arthropods. Bitrophic bioassay and tritrophic bioassay were conducted to evaluate the potential impact of Cry1Ac/Cry1Ab-expressing rice Huahui 1 and its non-transgenic counterpart Minghui 63 on fitness parameters of adult *M*. *discolor*. The results showed that the survival, and fecundity of this beetle’ adults were not different when they fed on Bt rice or non-Bt rice pollen or *Nilaparvata lugens* (Stål) reared on *Bt* rice or non-*Bt* rice. Toxicity assessment to ensure *M*. *discolor* adults were not sensitive to Cry1Ab or Cry1Ac protein independent from the pollen background, *M*. *discolor* adults were fed with an artificial diet containing Cry1Ac, Cry1Ab or both protein approximately 10 times higher concentration than in Huahui 1 rice pollen. No difference was detected for any of the life-table parameters tested between Cry protein-containing and pure diet. Artificial diet containing E-64 (N-(trans-Epoxysuccinyl)-L-leucine 4-guanidinobutylamide) was included as a positive control. In contrast, the pre-oviposition and fecundity of *M*. *discolor* were significantly adversely affected by feeding on E-64-containing diet. In both bioassays, the uptakes of Cry protein by adult *M*. *discolor* were tested by ELISA measurements. These results indicated that adults of *M*. *discolor* are not affected by Cry1Ab- or Cry1Ac-expressing rice pollen and are not sensitive to Cry protein at concentrations exceeding the levels in rice pollen in Huahui1. This suggests that *M*. *discolor* adults would not be harmed by Cry1Ac/Cry1Ab rice if *Bt* rice Huahui 1 were commercialized.

## Introduction

Rice, *Oryza sativa* L., is a main food crop for 3.5 billion people in the world [1]. Leaffolders such as *Chilo suppressalis* (Walker) (Lepidoptera: Pyralidae), *Scirpophaga incertulas* (Walker) (Lepidoptera: Pyralidae) and *Cnaphalocrocis medinalis* Guenée (Lepidoptera: Pyralidae) are three of the most serious pests of rice in temperate to tropical Asia [2–4]. Many synthetic *Bt* genes including *cry1Ab*, *cry1Ac* and *cry1B*, are derived from the bacterium *Bacillus thuringiensis* Berliner transferred into rice, conferring high resistance against lepidopteran pests have been developed since 1993 [4–6].

Huahui 1 is a transgenic *Bt* rice created by fusion a synthetic *cry1Ab*/*cry1Ac* gene into rice MingHui63 [7–8], and it is a lepidopteran resistant variety. From 2009 to 2014, Huahui 1 and its hybrid line *Bt* Shanyou 63 were issued a safety certificate and approved for limited release in farmers' fields in the Hubei Province in China [8–9].

*Micraspis discolor* is distributed from Southern China to Southern Asia [10–13]. It feeds on plants’ pollen, such as pollen of *Triticeae Dumort*, *Oryza sativa* L., as well as on a wide range of soft-bodied arthropods and eggs. It has been reported as both phytophagous and entomophagous. In rice ecosystems, adult *M*. *discolor* feed on rice pollen and *Nilaparvata lugens* (Stål) (BPH), *Sogatella furcifera* (Horváth) (WBPH), aphids and eggs or larvae of *C*. *suppressalis* and *T*. *incertulas etc*.

It has been recorded that planthoppers -BPH and WBPH are the predominant prey of *Micraspis* spp. [14–16], so *M*. *discolor* can play important roles in natural control of pest insects [10–14]. Feeding preference studies showed that adults of *M*. *discolor* have a significant preference for rice pollen over all insects from rice fields in paired-choice tests [14]. 1st and 2nd instar larvae mainly feed on rice pollen. *M*. *discolor* can complete its life on rice pollen or BPH alone [11].

Previous studies have demonstrated that *Bt* crop pollen expressing Cry1Ab, Cry3A and Cry3Bb1, poses little risk to predators [17–20]. As Huahui 1 may be commercialized in the future, the impact of *Bt* rice on *M*. *discolor* should be carefully evaluated before its commercialization. In this study, a comprehensive assessment of the effects of transgenic Cry1Ac/Cry1Ab rice Huahui 1 on adult *M*. *discolor* were investigated.

## Results

### The abundance of *Micraspis discolor* and other predatory Coleoptera in rice ecosystem in different rice growth periods in 3 rice-cropping seasons

**Fig 1:**
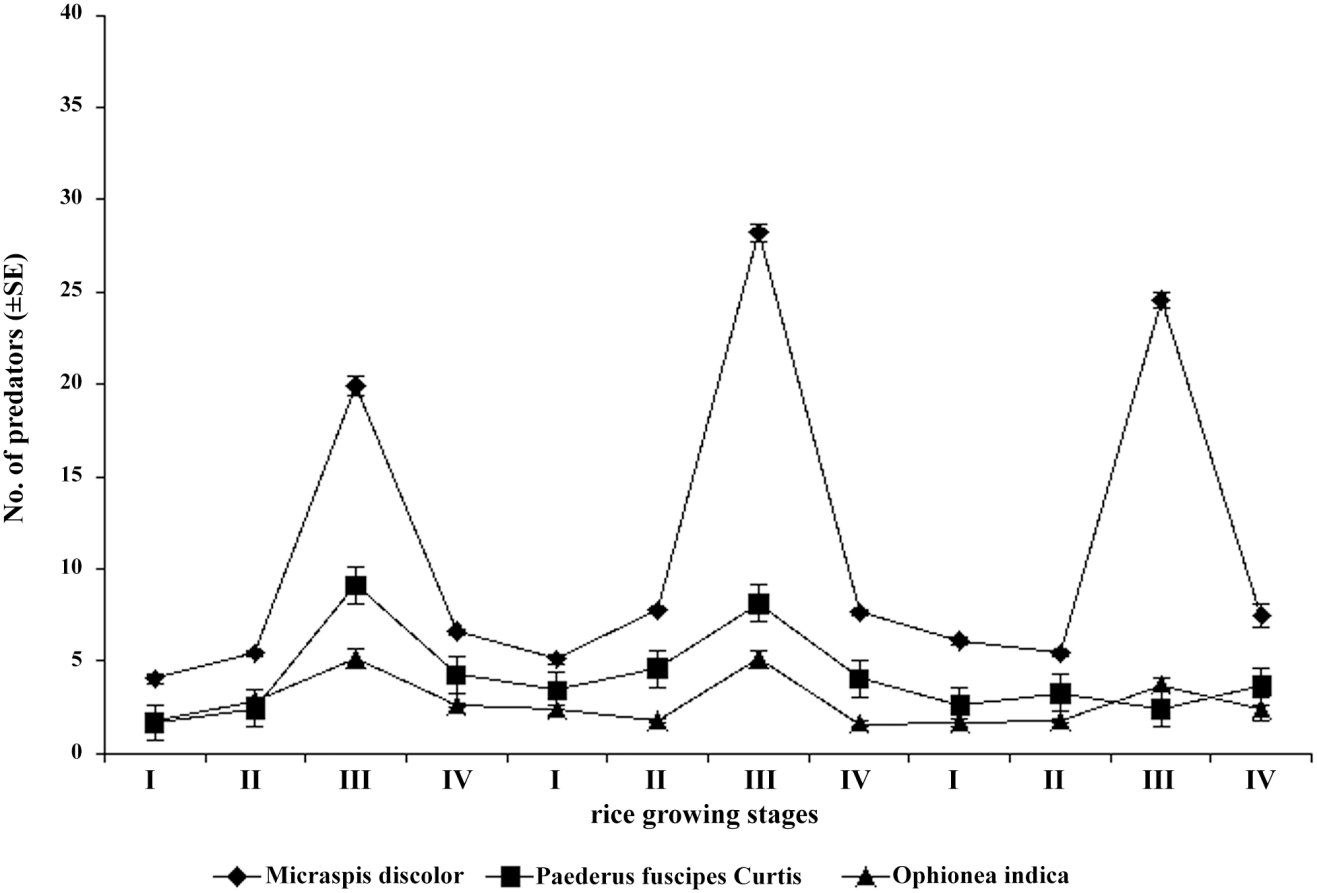
Different abundance of 3 predators in booting(I), tillering(II), early-blooming(III), blooming(IV) and filling(V) phases in Spring, Summer and Autumn rice seasons in 2013.

Field studies showed that there were same trends of the abundance change of coleoptera predators in rice ecosystem in 3 rice-cropping seasons in Minghui 63 paddies. 3 species of coleoptera predators, *M*. *discolor*, *Ophionea indica* (Thunberg), *Paederus fuscipes* Curtis were main predators which feed on soft-bodied insects in whole rice growth period. In Sanya rice ecosystem, adult *M*. *discolor* was the most dominant coleoptera predator in whole rice growth period especially in blooming stage in 3 rice seasons (Fig 1) (S1 File). Adults *M*. *discolor* were 19.85±2.20, 28.35±0.89, 24.55±1.73 in each replicate in early-blooming stage in Spring, Summer and Autumn rice seasons, 2–6 fold the quantities in booting, tillering and filling stage of 3 rice-cropping seasons.

### *Bt* rice pollen bioassay

**Fig 2:**
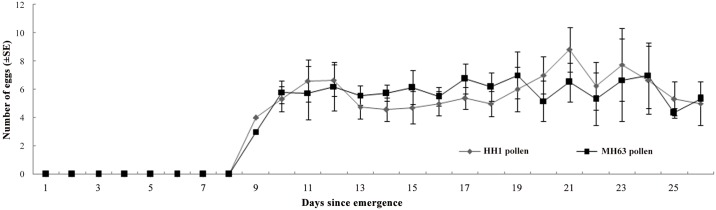
Mean (±SE) daily fecundity of *Micraspis discolor* fed pollen from Cry1Ab /Cry1Ac-expressing *Bt* rice or the corresponding non-transformed rice plants. N = 30.

Over 75% percent adult *M*. *discolor* survived the 26 days pollen feeding period, and the survival rates of males were slightly higher than that of females (Table 1) (S2 File). For both sexes, survival did not differ between the *Bt* and non-*Bt* pollen treatments. Similarly, the pre-oviposition period, total fecundity and female and male dry weight were not affected by the *Bt* pollen treatment (Table 1). Additionally, the daily fecundity of females was not significantly affected by feeding on Cry1Ac/Cry1Ab -expressing rice pollen (RM-ANOVA; F_1,76_ = 0.12, P = 0.74) (Fig 2) (S3 File).

**Table 1:** Impact of the consumption of pollen from Cry1Ab /Cry1Ac-expressing *Bt* rice or the corresponding non-transformed rice plant on different life-table parameters of adult *Micraspis discolor*. Data are provided as mean (±SE).

Fitness indicators	Minghui 63 pollen	Huahui 1 pollen	
Female survival (%)^a^	76.7(30)	80.0(30)	χ^2^ = 0.10 p = 0.75
Male survival (%)^a^	80.0(30)	83.3(30)	χ^2^ = 0.11 p = 0.74
Pre-oviposit. period (days) ^b^	13.17±0.08(30)	13.06±0.10(30)	U = 73787.5 p = 0.51
Fecundity (eggs/female) ^c^	19.97±0.37(30)	19.07±0.39(30)	t = 1.68 p = 0.09
Female adult dry weight (mg) ^c^	6.14±0.07(16)	6.26±0.06(17)	t = -1.38 p = 0.17
Female adult dry weight (mg) ^c^	3.97±0.07(17)	3.83±0.05(17)	t = 1.59 p = 0.12

The experiment was terminated after 26 days. N = 30.

^a^ Chi- square test

^b^ Mann- Whitney U test

^c^ Student’s t test

The ELISA measurements showed that the mean (±SE) concentration of Cry1Ab, Cry1Ac protein in the pollen was 15.26 ± 0.02μg /g and 11.92±0.01μg /g dry weight. No Cry1Ac or Cry1Ab protein was detected in any sample of non-*Bt* rice Minghui63 pollen.

When the experiment was terminated after 26 days, ELISA results showed that adults fed on *Bt* rice pollen contained Cry1Ac/Cry1Ab concentrations with a mean (±SE) of 2.09±0.01μg/g Cry1Ac and 1.55±0.02 μg/g Cry1Ab dry weight in female adults, 0.36±0.11μg/g Cry1Ac and 0.25±0.01μg/g Cry1Ab dry weight in male adults. No Cry toxin was detected in adults fed on non-*Bt* rice pollen.

### BPH reared on *Bt* rice or non-*Bt* rice bioassay

**Fig 3:**
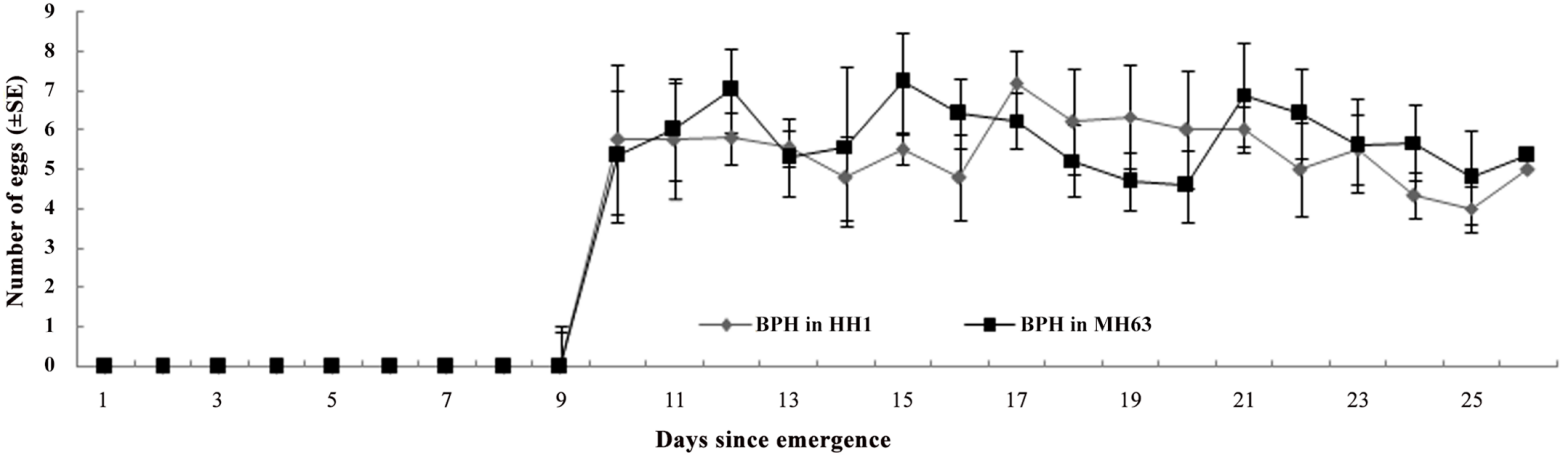
Mean (±SE) daily fecundity of *Micraspis discolor* fed BPH reared on Cry1Ab /Cry1Ac-expressing *Bt* rice or the corresponding non-transformed rice plant. N = 30.

Over 70% percent adult *M*. *discolor* fed BPH reared on *Bt* rice Huahui1 or Minghui 63 plants survived the 26 days period (Table 2) (S4 File). For both sexes, survival did not differ between BPH from *Bt* and non-*Bt* treatments. The pre-oviposition period, total fecundity and female and male dry weight were not different between 2 treatments (Table 2) (S4 File). The daily fecundity of females was not significantly affected by feeding on BPH from Huahui 1 rice (RM-ANOVA; F_1,64_ = 0.67, P = 0.42) (Fig 3) (S5 File).

**Table 2:** Impact of the consumption of BPH reared on Cry1Ab /Cry1Ac-expressing *Bt* rice or the corresponding non-transformed rice plants on different life-table parameters of adult *Micraspis discolor*. Data are provided as mean (±SE).

Fitness indicators	BPH on Minghui 63	BPH on Huahui 1	
Female survival (%)^a^	76.7(30)	73.3(30)	χ^2^ = 0.09 p = 0.77
Male survival (%)^a^	70.0(30)	73.3(30)	χ^2^ = 0.08 p = 0.77
Pre-oviposit. period (days) ^b^	13.13±0.08(30)	13.09±0.09(30)	U = 71149.0 p = 0.13
Fecundity (eggs/female) ^c^	18.40±0.74(30)	19.71±0.73(30)	t = -1.26 p = 0.21
Female adult dry weight (mg) ^c^	6.09±0.07(15)	6.25±0.06(16)	t = -1.70 p = 0.09
Female adult dry weight (mg) ^c^	3.91±0.07(15)	3.88±0.06(17)	t = 0.38 p = 0.71

The experiment was terminated after 26 days. N = 30.

^a^ Chi- square test

^b^ Mann- Whitney U test

^c^ Student’s t test

The ELISA measurements showed that the mean (±SE) concentration of Cry1Ab, Cry1Ac protein in the BPH reared on Huahui 1 was 3.23 ± 0.01μg /g and 2.46±0.01μg /g dry weight. No Cry1Ac or Cry1Ab protein was detected in any sample reared on non-*Bt* rice.

When the experiment was terminated after 26 days, ELISA results showed that female adults fed on BPH reared on *Bt* rice contained Cry1Ac/Cry1Ab concentrations with a mean (±SE) of 0.15±0.00μg/g Cry1Ab and 0.11±0.00μg/g Cry1Ac dry weight. Most male adults samples were detected no Cry1Ac/Cry1Ab concentration. No Cry toxin was detected in adults fed on BPH reared on non-*Bt* rice.

### Purified Cry toxin bioassay

**Fig 4:**
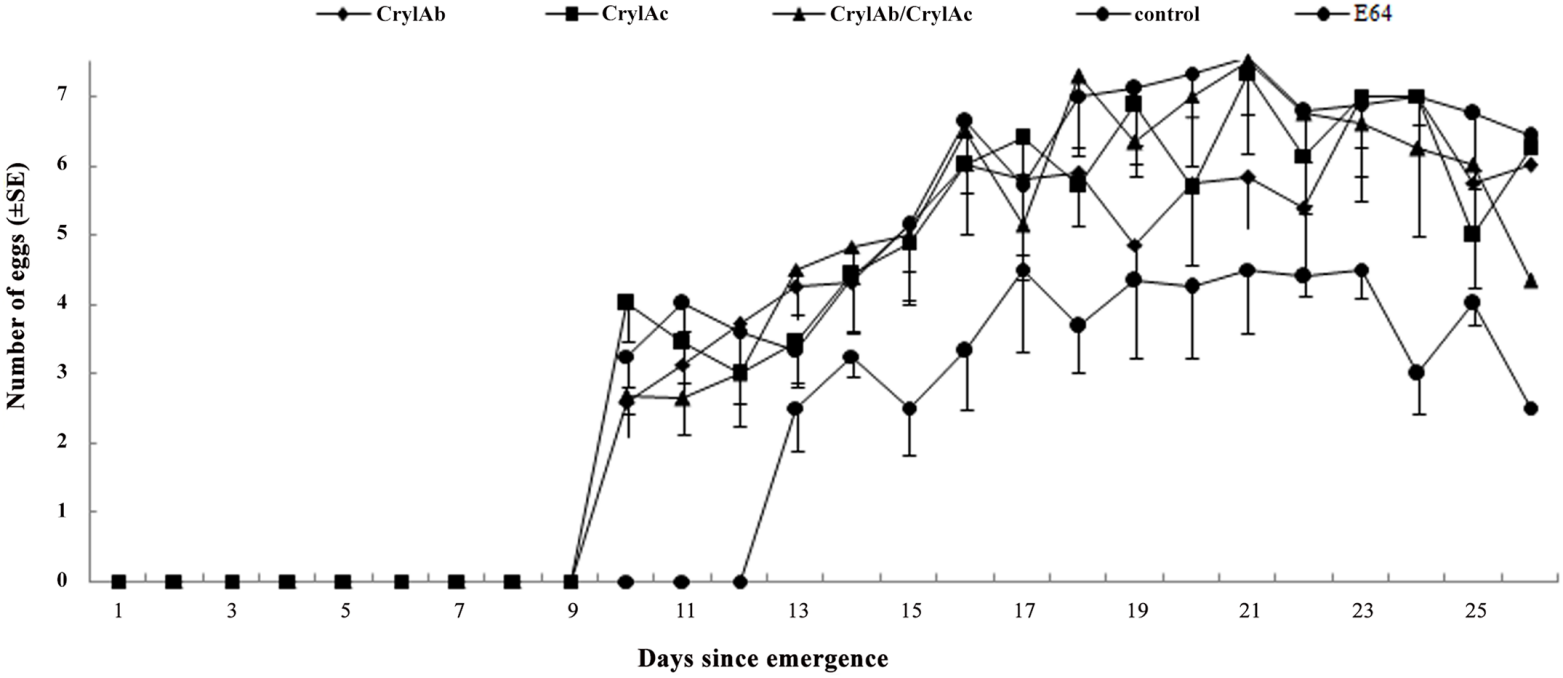
Mean (±SE) daily fecundity of *Micraspis discolor* fed an artificial diet containing 150 mg/g Cry1Ab, 120 mg/g Cry1Ac, 150 mg/g Cry1Ab and 120 mg/g Cry1Ac or 25 mg/g E-64(positive control) per g dry weight of artificial diet. Pure artificial diet served as a negative control.

The survival rates of adult *M*. *discolor* were above 65% and did not differ between adults feeding on pure artificial diet or any Cry toxin treatment (Chi-square test, P> 0.05) (Table 3) (S6 File). While consumption of diet containing E-64 caused a decline in adult survival by more than 10%, this difference also was not significant (χ2 = 1.36, P = 0.24) (Table 3) (S6 File). No significant differences were detected between the Cry1Ac/Cry1Ab treatment and the control for the pre-oviposition period, total fecundity and adult dry weight (all P > 0.05) (S6 File). In contrast, the pre-oviposition period was significantly prolonged (Mann—Whitney U test; U = 58347.5, p = 0), the total fecundity was significantly reduced (Dunnett test; P < 0.05) when *M*. *discolor* adults were fed with the E-64-containing diet compared with those fed with pure artificial diet (Table 3) (S6 File). Likewise daily fecundity did not differ between the Cry1Ab, Cry1Ac or Cry1Ab/ Cry1Ac with control treatments (P>0.05), while E-64 caused a significant reduction in daily fecundity (RM-ANOVA; F_1,49_ = 54.364, P < 0.01) (Fig 4) (S7 File).

**Table 3:** Effect of feeding a pure artificial diet or artificial diet with purified Cry1Ab, Cry1Ac and E-64 provided on different life-table parameters of adult *Micraspis discolor*.

Fitness indicators	Cry1Ab	Cry1Ac	Cry1Ab/Cry1Ac	control	E-64
Survival(%)^a^	80.6(31)	79.7(32)	75.9(29)	78.4(37)	65.5(29)
Pre-oviposit. Period(days) ^b^	13.87±0.70(31)	13.84±0.64(32)	13.62±0.62(29)	13.92±0.48(37)	16.79±0.51(29)**
Fecundity (eggs/female) ^c^	18.77±1.52(31)	19.44±1.60(32)	19.31±1.55(29)	20.86±1.41(37)	6.79±0.61(29) **
Female adult dry weight (mg) ^c^	6.30±0.18(14)	6.34±0.21(14)	6.16±0.28(13)	6.43±0.17(14)	6.09±0.19 (13)
Male adult fresh weight (mg) ^c^	4.03±0.16(14)	3.99±0.19(14)	3.97±0.16(13)	3.99±0.18(14)	3.81±0.15(13)

35–39 pairs of adult *Micraspis discolor* were fed an artificial diet containing 150 mg/g Cry1Ab, 120 mg/g Cry1Ac, 150 mg/g Cry1Ab and 120 mg/g Cry1Ac or 25 mg/g E-64 (positive control) per g dry weight of artificial diet. Pure artificial diet served as a negative control. Pairs producing no eggs were removed from the analyses.

Statistical comparisons were made between each of the Cry1Ab, Cry1Ac, Cry1Ab/ Cry1Ac and E-64 treatments with the control. Asterisks denote significant differences:

** p < 0.01

^a^ Chi-square test

^b^ Mann-Whitney U-test

^c^ Dunnett test

After 26 days diet feeding, the ingestion of Cry1Ac/Cry1Ab by adult *M*. *discolor* was confirmed by ELISA. Similar to Bt rice pollen feeding, the females contained much higher mean (±SE) concentrations of the protein (Cry1Ab 2.55 ± 0.02 μg/g, Cry1Ac 1.89± 0.03 μg/g dry weight) than males (Cry1Ab 0.41 ± 0.01μg/g and Cry1Ac 0.31 ± 0.02μg/g dry weight). No *Bt* protein was detected in adults fed pure artificial diet.

## Discussion

New high yielding varieties, pesticides and nitrogen fertilizers have doubled the production of many crops including rice [21–22]. But the inputs of insecticides and fertilizers have resulted in some negative effects on environments [23–25]. Current methods for controlling insect pests mainly include good farming practices, biological control, breeding and growing resistant varieties, and the use of pesticides. However, the application of chemical insecticide is the major control measure for rice insect pests in Asia, which not only causes severe environmental pollution and the resurgence of herbivores but also reduces populations of the natural enemies of herbivores [23–25].

The Cry1Ab/Cry1Ac protein expressed *Bt* rice Huahui 1 was issued a safety certificate in China in 2009. It is lepidopteran specific with high activity against *C*. *suppressalis*, *S*. *incertulas* and many other lepidopteran pests [5], [26–28]. Although predators were not the target insects, *M*. *discolor* may have been altered due to the introduction of Cry1Ab/Cry1Ac gene. Such a potential effect requires proper assessment of the ecological risks posed by *Bt* rice, by carrying out a combination of laboratory tests, field experiments and longer-term monitoring, as suggested by Jepson *et al*. [29].

In the laboratory study, ELISA measurements showed that *Bt* rice Huahui 1 pollen contained high concentrations of Cry1Ab/Cry1Ac, and Cry proteins also were detected in *M*. *discolor* when fed *Bt* rice pollen, although the *Bt* protein levels were 7 times lower for Cry1Ab and 6 times lower for Cry1Ac compared to the concentrations in the *Bt* rice pollen. The *Bt* protein levels of BPH were much lower than that in *Bt* rice pollen, the tritrophic results of BPH was the same as Huahui 1 pollen feeding bioassay. Same reports have been reported from other bitrophic and tritrophic experiments in which *Bt* plants pollen was used to expose predators to the insecticidal compounds [17, 20, 29–31].

The pollen/BPH feeding bioassays showed no adverse effect on the fitness of adult *M*. *discolor* after ingestion of *Bt* rice Huahui 1 pollen/BPH expressing Cry1Ab/ Cry1Ac compared with pollen from the corresponding nontransformed rice Minghui 63. Similarly no negative effects on the survival and reproduction of adult *M*. *discolor* were observed when this beetles were provided with an artificial diet containing Cry1Ab, Cry1Ac or Cry1Ab/ Cry1Ac at a concentration that was approximately 10 times higher than that measured in the *Bt* rice pollen. E-64 treatment (positive control) caused a significant prolongation of the pre-oviposition period and a decrease in fecundity. E-64 has been used as a positive control in insect bioassays for toxicity assessments, such as *Coleomegilla maculata* [32–33].

Our study indicated that *Bt* rice pollen or BPH reared on *Bt* rice has no adverse effect on *M*. *discolor* which fed on rice pollen or BPH alone. This result agrees with the studies of Wang *et al*., adults of *Chrysoperla*. *sinica* are not affected by Cry2Aa-expressing rice pollen and are not sensitive to Cry2Aa protein at concentrations exceeding the levels in pollen [34]. Bai *et al*. also reported Cry1Ab toxin expressed in *Bt* rice pollen had no evident negative impacts on *Propylea japonica* fitness when the pollen was used as a food by this beetle [26], Other rice crop also showed the same results. Li *et al*. reported adults of *Chrysoperla carnea* (Stephens) are not affected by *Bt* maize pollen and *Adalia bipunctata* are not sensitive to Cry1Ab and Cry3Bb1 at concentrations exceeding the levels in pollen [35–36]. Li *et al*. also reported Cry1Ac and Cry2Ab *Bt* cotton demonstrates no detrimental effects on *Coleomegilla maculata* [32–33].

The E-64 treatment (positive control) caused a significant prolongation of the pre-oviposition period and a decrease in fecundity. Similar trend was observed for female *M*. *discolor*. These results demonstrate that the experimental system was able to detect treatment effects if present and is suitable for assessing the hazards of orally active insecticidal compounds on adult *M*. *discolor*. E-64 has been used as a positive control in insect bioassays for toxicity assessments, namely for *Coleomegilla maculate* [32–33] and *Coccinella septempunctata* [37].

In summary, this study using bitropic and tritropic bioassays and a direct feeding bioassay provides the most complete information so far on the potential direct and indirect effects of Cry1Ab and Cry1Ac on the ladybird beetle, *M*. *discolor*, a common and abundant predator in rice systems. ELISA measurements and sensitive-insect bioassays confirmed that the test insects were exposed to high concentrations of biologically active Cry proteins throughout the duration of the bioassays. This suggests that *M*. *discolor* adults would not be harmed by Cry1Ab/Cry1Ac rice if transgenic *Bt* rice Huahui 1 were commercialized.

Reports showed that *Bt* rice Shanyou 63 which was produced by crossbreeding Zhenshan 97A with Huahui 1 reduced pesticide applications 80% [38]. Meanwhile *Bt* rice Huahui 1 does not affect a important predator *M*. *discolor* adults. Consequently, Huahui 1 would not pose negative effects on both environment and predators if it were commercialized.

## Materials and Methods

### Ethics Statement

No specific permits were required for the described studies.

### Plant materials

Transgenic Bt Huahui 1 and its non-transgenic counterpart Minghui 63 were evaluated.

### Insects

*M*. *discolor* and *Nilaparvata lugens*(Stal) were collected from rice fields in the experimental farm of Sanya Science&Technology Academy for crop winter multiplication during May 2012 and since then maintained in the laboratory without introductions of field-collected insects. Eggs collected from the colony were kept in a climatic chamber at 16:8 h (L: D), 28±1°C, and 75 ± 5%. After hatching, the larvae were kept individually in glass tubes (2 cm diameter, 9 cm long). 5–15 Drosophila adults were supplied daily to the tubes. After 6 generation newly emerged (<24 h after emergence) adults *M*. *discolor* were used for the experiments.

*N*. *lugens* maintained in rice Yuzhenxiang seedlings in the greenhouse in the experimental base of Tropical biological technology insititute in Wenchang couty. After more than 20 generation *N*. *lugens* were transferred to Huahui1 and Minghui 63 seedlings, 3rd instar *N*. *lugens* from Huahui1 and Minghui 63 were used for the experiments.

### Purified proteins

Cry1Ab toxin and Cry1Ac toxin (99%) used in this study was provided by EnvirologixInc. E-64(advanced pure) was provided by the Amresco Company.

### The abundance of Coleoptera predators in rice ecosystem

There are three rice-cropping seasons according to sowing date in the studied area. The non-transgenic control Minghui 63 were sowed in early December in 2012, early April in 2013 and early July in 2013, and transplanted 1 month later. Field insect sampling by the D-vac method in minghui63 field were conducted at the experimental farm of Sanya Science&Technology Academy for crop winter multiplication in Hainan Province, China in 2013. Five samples were taken from each plot. For taking a sample, a plastic bin covered 20x20 cm^2^ was placed over one hill and all arthropods inside the bin were carefully collected with a blower-vac machine sampler. There were 3 replicates, and each plot was 400 m^2^. Rice seedlings were transplanted by hand at a density of 3 seedlings per hill. Spacing between rows was 20 cm and hills within rows were spaced 15 cm apart.

Field insect sampling were conducted in rice tillering, booting, early-blooming, blooming and filling stage with five point sampling method in every rice seasons. Each sampling site contained 9 rice hills. All treatments were managed by normal cultural practices except for pesticide sprays. Collected insects were put in 80% alcohol, and Coleoptera predators were counted in laboratory.

### Rice pollen

Pollen was obtained from greenhouse-grown rice of the *Bt* rice lines Huahui 1 and the nontransgenic line Minghui 63 in the Wenchang experimental base of Tropical biological technology insititute.

In the morning, inflorescences were cut from plants before anthesis. The obtained pollens was placed in sealed plastic vials and kept at—20°C until use. In the laboratory, anthers enclosed within glumes were striped off by scissors.

### *Bt* rice pollen bioassay and BPH bioassay

The experiment was conducted in the climatic carbine with photoperiod 16:8 h (L: D), 28±1°C, and 75 ± 5% RH.

For each treatment, new emergenced adults were transferred to a Petri dish (10.0 cm diameter, 2.6 cm tall). After 3 days, adults were paired and placed in glass tubes (2cm diameter, 9cm long) containing sufficient *Bt* or non-*Bt* rice pollens or BPH reared on Huahui1 or Minghui 63 and a water-saturated cotton ball with 5% leechee honey. Thereafter, daily fecundity and adult survival were recorded every 6 h. After 26 days, dry male and female adults were weighed.

When egg clutches were observed, adults *M*. *discolor* as well as the rice pollens/BPH were transferred to other clear glass tubes, and the numbers of egg clutches were recorded.

### Purified toxin bioassay

The purified toxin bioassay was carried out as described above. The experimental system was used to assess the toxicity of Cry1Ac and Cry1Ab on *M*. *discolor*. Five diet treatments were tested: 1) artificial diet (negative control) containing of litchi honey, egg yolk powder (in proportions 6:3); 2) artificial diet containing Cry1Ab at 150 mg/g weight of diet; 3) artificial diet containing Cry1Ac at 120 mg/g dry weight of diet; 4) artificial diet containing Cry1Ab at 150 mg/g and Cry1Ac at 120 mg/g dry weight of diet; 5) artificial diet containing E-64 at 25 mg/g fresh weight of diet (positive control). Diets were replaced every day. The experiment was conducted with 30–33 pairs neonate *M*. *discolor* adults per treatment. The insects were observed every 6 h and their survival and development were recorded.

### ELISA measurements

The concentrations of Cry1Ab and Cry1Ac in rice pollen, adult *M*. *discolor* and BPH were measured by enzyme-linked immunosorbent assays (ELISA) using the Cry1Ab/Cry1Ac detection kits from Envirologix (Portland Maine, USA). Prior to analysis, insects were washed in phosphate buffered saline with Tween-20 (PBST) buffer (provided in the kit) to remove any *Bt* toxin from their outer surface.

After adding PBST to the samples at a ratio of at least 1:10 (mg sample:ml buffer) in 1.5 ml centrifuge tubes, the samples were fully ground by hand using a plastic pestle. After centrifugation and appropriate dilution of the supernatants, ELISA was performed according to the kit’s instructions. The measured OD values were calibrated to a range of concentrations of Cry1Ab and Cry1Ac made from purified toxin solution.

### Data analysis

For the pollen bioassay, statistical analyses were conducted between the *Bt* pollen and non-Bt pollen treatments. For the BPH bioassay, statistical analyses were conducted between BPH from *Bt* rice Huahui 1 and non-*Bt* rice Minghui 63 treatments. In the purified toxin experiment, comparisons were made between the Cry1Ac, Cry1Ab, Cry1Ac /Cry1Ab or E-64 treatment with control (pure artificial diet). For both experiments, Chi-square tests were carried out to compare adult survival (females and males separately). Mann—Whitney U-tests were used to compare the preoviposition. Data on the total fecundity and adult dry weight were compared using Student’s t-tests in the pollen feeding bioassay and by Dunnett tests in the purified protein bioassay. Daily fecundity data were analyzed using repeated measures (RM) ANOVA.

All statistical analyses were conducted using the software package SPSS (version 16; SPSS, Inc., Chicago, IL).

## Supporting Information

S1 File Different abundance of 3 predators in booting(I), tilling(II), early-blooming(III), blooming(IV)and filling(V) phase in Spring, Summer and Autumn rice seasons in 2013 (Fig 1). (XLS)

S2 File Impact of the consumption of pollen from Cry1Ab /Cry1Ac-expressing *Bt* rice or the corresponding non-transformed rice plant on different life-table parameters of adult *Micraspis discolor* (Table 1). (XLS)

S3 File Daily fecundity of *Micraspis discolor* fed pollen from Cry1Ab /Cry1Ac Bt rice or the corresponding non-transformed rice plants (Fig 2). (XLS)

S4 File Impact of the consumption of BPH from Cry1Ab /Cry1Ac Bt rice or the corresponding non-transformed rice plants on different life-table parameters of adult *Micraspis discolor* (Table 2). (XLS)

S5 File Daily fecundity of *Micraspis discolor* fed BPH from Cry1Ab /Cry1Ac Bt rice or the corresponding non-transformed rice plant (Fig 3). (XLS)

S6 File Effect of feeding a pure artificial diet or artificial diet with purified Cry1Ab, Cry1Ac and E-64 provided on different life-table parameters of adult *Micraspis discolor* (Table 3). (XLS)

S7 File Daily fecundity of *Micraspis discolor* fed an artificial diet containing 150 mg/g Cry1Ab, 120 mg/g Cry1Ac, 150 mg/g Cry1Ab and 120 mg/g Cry1Ac or 25 mg/g E-64(positive control) per g dry weight of artificial diet (Fig 4). (XLS)

## References

[1] Singh MK, Prasad SK. Agronomic Aspects of Zinc Biofortification in Rice (Oryza sativa L.) Proceedings of the national academy of sciences,India section B:biological Sciences. 2014; 84(3): 613–623.

[2] Dale D. Insect pests of the rice plant—their biology and ecology: stem borers In: Heinrichs EA (ed) Biology and management of rice insects. Wily Eastern, New Delhi; 1994 pp 388–408.

[3] Sheng CF, Xuan WJ, Jiao XG, Su JW, Shao QC, Song FB. Causes, trend and control strategies of disaster by rice borers in China. J. Nat. Disasters. 2002; 11: 103–107 (in Chinese with English abstract).

[4] Chen XN, Wu JC, Ma F. Brown Planthopper: Occurrence and Control. China Agriculture Press, Beijing, China; 2003 pp 1–2 (in Chinese).

[5] Shu QY, Ye QY, Cui HR, Cheng XY, Xiang YB, Wu DX, et al Transgenic rice plants with a synthetic cry1Ab gene from *Bacillus thuringiensis* were highly resistant to eight lepidopteran rice pest species. Mol Breeding. 2000; 6:433–439.

[6] Cohen MB, Chen M, Bentur JS, Heong KL, Ye GY. Bt rice in Asia: potential benefits, impact, and sustainability In: Romeis J., Shelton A.M., Kennedy G.G. (Eds.), Integration of Insect-Resistant Genetically Modified Crops with IPM Systems. Springer, Dordrecht, The Netherlands 2008; 223–248.

[7] Tu J, Zhang G, Datta K, Xu C, He Y, Zhang Q, et al Field performance of transgenic elite commercial hybrid rice expressing *Bacillus thuringiensis* d-endotoxin. Nat. Biotechnol. 2000; 18, 1101–1104. 11017051 10.1038/80310

[8] Wang EH, Yu Z, Hu J, Jia XD, Xu HB. A two-generation reproduction study with transgenic Bt rice TT51 in Wistar rats. Food and Chem Toxico. 2014; 65, 312–320. 10.1016/j.fct.2013.11.045 24309144

[9] Tian JC, Liu ZC, Chen M, Chen Y, Chen XX, Peng YY, et al Laboratory and field assessments of prey-mediated effects of transgenic Bt rice on Ummeliata insecticeps (Araneida: Linyphiidae). Environ Entomol. 2010; 39: 1369–1377. 10.1603/EN10003 22127189

[10] Ministry of Agriculture of the People's Republic of China (MAPRC) (2009) The second list of approval agricultural genetically modified organisms' safety certificates in 2009. http://www.stee.agri.gov.cn/biosafety/spxx/P020091127591594596689.pdf. [In Chinese]. Accessed: 15 March 2012.

[11] Zhang SM, Jiang YC. Xue FS. Study on Micraspis discolor (Fabricius). Nautral enemies of insects. 1982; 4(3):28–31 (in Chinese).

[12] Jiang YC. Shu ZM. The feeding habits and classification of Micraspis discolor (Fabricius). Acta Entomologica Sinica. 1985; 28(1):115–117.

[13] Jiang YC. The biological characteristics and the protection of Micraspis discolor (Fabricius) Entomological Knowledge. 1995; 32(2):114–115 (in Chinese).

[14] Pushpendra KS, Prakash CJ. New Records of Coccinellid Beetles (Coccinellidae: Coleoptera) from District Dehradun, (Uttarakhand), India New York Science Journal. 2010; 3(6):112–120.

[15] Shanker C, Mohan M, Sampathkumar M, Lydia Ch, Katti G. Functional significance of Micraspis discolor (F.) (Coccinellidae: Coleoptera) in rice ecosystem. J Appl Entomol. 2013; 137: 601–609.

[16] Rattanapun W. Biology and potentiality in biological control of Micraspis discolor (Fabricius) (Coleoptera: Coccinellidae). Commun Agric Appl Biol Sci. 2012; 77(4):541–548. 23885421

[17] Begum MA, Jahan M, Bari MN, Hossain MM, Afsana N. Potentiality of *Micraspis discolor* (F.) as a Biocontrol Agent of *Nilaparvata lugens* (Stal). J of Biol Sci. 2002; 2(9): 630–632.

[18] Pilcher CD, Obrycki JJ, Rice ME, Lewis LC. Preimaginal development, survival, and field abundance of insect predators on transgenic Bacillus thuringiensis corn. Env Entomol. 1997; 26:446–454.

[19] Riddick EW, Barbosa P. Impact of Cry3A-intoxicated Leptinotarsa decemlineata (Coleoptera: Chrysomelidae) and pollen on consumption, development, and fecundity of Coleomegilla maculata (Coleoptera: Coccinellidae). Ann Entomol Soc Am. 1998; 91:303–307.

[20] Duan JJ, Head G, McKee MJ, Nickson TE, Martin JW, Sayegh FS. Evaluation of dietary effects of transgenic corn pollen expressing Cry3Bb1 protein on a non-target ladybird beetle, *Coleomegilla maculata*. Entomol Exp Appl. 2002; 104:271–280.

[21] Conway GR. The Doubly Green Revolution: food for all in the 21st Century. Cornell University Press New York 1997.

[22] Conway GR, Pretty GN. Unwelcome harvest: Agriculture and pollution. Earthscan publications Ltd London 1991.

[23] Huang SN, Hu R, Rozelle S, Pray C. Insect-resistant GM rice in farmers’ fields: assessing productivity and health effects in China. Science. 2005; 308:688–690. 15860626 10.1126/science.1108972

[24] Lou YG, Zhang GR, Zhang WQ, Hu Y, Zhang Q. Reprint of: Biological control of rice insect pest in China. Biol Control. 2014; 68:103–116.

[25] Tanaka K, Endo S, Kazano H. Toxicity of insecticides to predators of rice planthoppers:spides, the mired bug and the dryinid wasp. Appl Entomol Zool. 2000; 35:1177–1187.

[26] Bai YY, Jiang MX, Cheng JA. Effects of transgenic cry1Ab rice pollen on fitness of Propylea japonica (Thunberg). J Pest Sci. 2005; 78: 123–128.

[27] Wu G, Cui HR, Shu QY, Ye GY, Xie XB, Xia YW, et al Expression patterns of cry1Ab gene in progenies of ‘‘Kemingdao” and the resistance to striped stem borer. Scientia Agricultura Sinica. 2001; 34:496–501.

[28] Ye GY, Shu QY, Yao HW, Cui HR, Cheng XY, Hu C, et al Field evaluation of resistance of transgenic rice containing a synthetic cry1Ab gene from *Bacillus thuringiensis* Berliner to two stem borers. J Econ Entomol. 2001; 94:271–276. 11233125 10.1603/0022-0493-94.1.271

[29] Jepson PC, Croft BA, Pratt GE. Test systems to determine the ecological risks posed by toxin release from *Bacillus thuringiensis* genes in crop plants. Mol Ecol. 1994; 3:81–89.

[30] Sims SR. Bacillus thuringiensis var. kurstaki (CryIA (C)) protein expressed in transgenic cotton: effects on beneficial and other non-target insects. SW Entomol. 1995; 20:493–500.

[31] Cui JJ, Xia JY. Effect of transgenic Bt cotton on the population dynamics of natural enemies. Acta Gossypii Sinica. 1999; 11:84–91.

[32] Li YH, Romeis J, Wang P, Peng YF, Shelton AM. A comprehensive assessment of the effects of Bt cotton on Coleomegilla maculata demonstrates no detrimental effects by Cry1Ac and Cry2Ab. PLoS One. 2011; 6(7): e22185 10.1371/journal.pone.0022185 21765949 PMC3134477

[33] Li YH, Ostrem J, Romeis J, Hellmich RL, Chen M. Development of a Tier-1 assay for assessing the toxicity of insecticidal substances against *Coleomegilla maculata*. Environ Entomol. 2011; 40:496–502.

[34] Wang YY, Li YH, Romeis J, Chen XP, Zhang J, Chen HY, et al Consumption of Bt rice pollen expressing Cry2Aa does not cause adverse effects on adult Chrysoperla sinica Tjeder(Neuroptera:Chrysopidae). Biol Control. 2012; 61(3):246–251.

[35] Li YH, Meissle M, Romeis J. Consumption of Bt Maize Pollen Expressing Cry1Ab or Cry3Bb1 Does Not Harm Adult Green Lacewings, Chrysoperla carnea (Neuroptera: Chrysopidae). Plos One. 2008; 3(8):e2909 10.1371/journal.pone.0002909 18682800 PMC2488376

[36] A′lvarez-Alfageme F, Bigler F, Romeis J. Laboratory toxicity studies demonstrate no adverse effects of Cry1Ab and Cry3Bb1 to larvae of Adalia bipunctata (Coleoptera: Coccinellidae): The importance of study design. Transgenic Res. 2011; 20: 467–479. 10.1007/s11248-010-9430-5 20740377 PMC3090567

[37] A′lvarez-Alfageme F, Pa′linka′s Z, Bigler F, Romeis J. Development of an early-tier laboratory bioassay for assessing the impact of orally-active insecticidal compounds on larvae of Coccinella septempunctata (Coleoptera: Coccinellidae). Environ Entomol. 2012; 41: 1687–1693. 10.1603/EN12032 23321119

[38] Wang YM, Zhang GA, Du JP, Liu B, Wang MC. Influence of transgenic hybrid rice expressing a fused gene derived from *cry 1Ab* and *cry 1Ac* on primary insect pests and rice yield. Crop Prot. 2010; 29,128–133.

